# Overexpression of Chromatin Assembly Factor-1/p60 helps to predict the prognosis of melanoma patients

**DOI:** 10.1186/1471-2407-10-63

**Published:** 2010-02-24

**Authors:** Massimo Mascolo, Maria Luisa Vecchione, Gennaro Ilardi, Massimiliano Scalvenzi, Guido Molea, Maria Di Benedetto, Loredana Nugnes, Maria Siano, Gaetano De Rosa, Stefania Staibano

**Affiliations:** 1 Department of Biomorphological and Functional Sciences, Pathology Section, University of Naples "Federico II", School of Medicine, Naples, Italy; 2 Department of Systematic Pathology, Section of Dermatology, Allergology and Venereology, University of Naples "Federico II", School of Medicine, Naples, Italy; 3 Department of Systematic Pathology, Section of Plastic Surgery, University of Naples "Federico II", School of Medicine Naples, Italy; 4 Department of Medicine, University of Naples "Federico II", School of Medicine, Naples, Italy; 5 Cooperative Melanoma Intergroup, University of Naples "Federico II", School of Medicine, Naples, Italy; 6 CROB, Oncology Research Center of Basilicata, Rionero in Vulture, Potenza, Italy

## Abstract

**Background:**

Cutaneous melanoma (CM) is the most lethal form of skin malignancy, which registers a constant increase in incidence worldwide. The identification of molecular alteration(s) involved in its biological aggressiveness represents a major challenge for researchers, considering that existing therapies are ineffective to treat metastasizing cases. The epigenetic control of chromatin dynamics during DNA synthesis, replication, and repair is fundamental for the orderly progression of cell proliferation. The Chromatin Assembly Factor 1 (CAF-1) complex acts as a major regulator of this process; its intermediate (p60) subunit has been recently proposed as a novel proliferation and prognostic marker for several tumors. We aimed to establish if the evaluation of the expression of CAF-1/p60 in primary CM may help define the prevision of outcome of patients.

**Methods:**

Immunohistochemistry with anti-CAF-1/p60 was performed on paraffin-embedded tissue sections of 130 cases of primary CM retrieved from the archive files of the Department of Biomorphological and Functional Sciences, Section of Pathology, University "Federico II" of Naples, Italy. Results were compared with histopathological and follow-up data of patients.

**Results:**

CAF-1/p60 was expressed in all CM. A significant statistical association between the overexpression of the protein and the occurrence of skin, node and/or distant metastases (P < 0.05) emerged, independently from histopathological prognostic factors.

**Conclusions:**

CAF-1/p60 looks promising as a new prognostic marker for CM and sheds new light on the molecular events associated with photocancerogenesis and melanoma biology.

The screening for CAF-1/p60 might contribute to the molecular sub-classification of CM, with improved translational outcomes.

## Background

The incidence of cutaneous melanoma (CM) has shown one of the higher increases of any form of cancer during the past two generations [1]. Worldwide, about 100.000 new cases of CM are diagnosed each year and account for more than 70% of all the deaths from skin tumors [1-5].

The increase in CM incidence is shared by Australia, New Zealand, United States and most of European States, notwithstanding the better knowledge of environmental and phenotypic risk factors and ongoing primary and secondary prevention strategies [6-9]. To date, according to the updated AJCC staging system, the most reliable predictors of patient outcome remain the extent of infiltration (Breslow thickness), the presence of ulceration and, to a lesser extent, the tumor proliferation index [10]. The estimated 10-year survival rates for non-metastatic CM range from 93% for patients with "thin" tumors (</= 1.0 mm of dermal invasion, without ulceration and/or mitotic index < 1/mm^2^, Stage Ia), to 39% for patients with ulcerated tumors deeper than 4.0 mm [10]. In about two-third of progressing CM, loco-regional (skin and/or lymphatic) metastatic spreading occurs, whereas in the remaining cases, haematogenous metastases arise directly, at distant anatomic sites [11].

Recent improvements in diagnostic techniques (i.e.: epiluminescence dermoscopy) have led to diagnose most CM at an early stage. However, the number of patients that die for the disease remains still substantially unchanged.

This disappointing result could be explained, at least in part, by the heterogeneous nature of this malignancy [5]. At present, it is thought that the aggressiveness of melanoma may be conditioned by the variable concurrence of multiple factors, intrinsic to neoplastic cells (i.e., imbalance of cell proliferation and apoptosis control pathways), and/or micro-environmental (i.e., degree of tumor neo-angiogenesis, expression of adhesion molecules facilitating the migration of malignant cells) [12].

As a result, CM may ultimately show a biological behavior often unpredictable by means of the classical histological prognostic parameters [10].

"Thin" melanomas can metastasize early, while some "thick" tumors may show only late metastasis ("dormant" melanoma) [13].

At present, except for high-dose IFN as adjuvant therapy for stage III disease, effective strategies to treat metastasizing melanomas are lacking; the median survival of patients with distant metastases ranges from 6 to 10 months, with a 5-years survival lesser than 5% [14]. Therefore, the attention of the melanoma research community is devoted to the chance of translate significant molecular studies results on melanoma biology into clinical correlates and new therapeutic agents directed at specific pathways.

Proteomic and genomic studies have revealed, in CM, alterations of the expression of many oncogenes and tumor suppressor genes responsible of either the DNA damage repair and cell cycle control [15-19].

A fundamental role in the regulation of both these processes has emerged for the molecular factors responsible of the epigenetic regulation of nuclear chromatin dynamics, that control also the packaging and interpretation of the genome, in response to environmental stimuli [20-24].

Histone chaperones play a pivotal role in this process. They drive the incorporation of different histones into DNA to the sites where nuclear chromatin has to be newly formed or remodeled [25].

Among these, the Chromatin Assembly Factor-1 (CAF-1), a trimeric protein complex formed by the p48, p60, and p150 subunits, promotes histone incorporation into chromatin and acts in strict association with both the S-phase and DNA repair [26-29].

CAF-1 ensures the restoration of chromatin structure, providing that all aspects, including nucleosome position and epigenetic imprints, are re-established during the final step of DNA-repair, before cell-replication.

Recently, CAF-1/p60 has been proposed as a new proliferation and prognostic marker, since it has been found over-expressed in a series of human malignancies, in close association with their biological aggressiveness [30-32].

Starting from these postulates, we sought to examine the immunohistochemical expression of CAF-1/p60 on a selected series of primary CM. These tumors notoriously look at UV-radiation as a major environmental pathogenetic factor, and are characterized, in metastasizing cases, by an extreme deregulation of cell proliferation and defects in DNA-repair. We have previously evaluated the expression of the poly(ADP-ribose)polymerase 1 (PARP 1) in a series of melanomas of photo-exposed skin areas [18]. As it is known, the family of PARP proteins is directly involved in the epigenetic control of DNA-repair and cell death triggered by DNA damage. We found a significative statistical correlation between the inhibition of the PARP-1 mediated apoptosis and DNA repair process, and the metastasizing behavior of CM [18]. In addition, we hypothesized that the imbalance of PARP-1 activity could also be responsible, at least in part, for the chemoresistence of metastatic CM [18].

In the present study, considering the key role of CAF-1 in the epigenetic regulation of either cell proliferation and DNA-repair, we focused on the expression of CAF-1/p60 on a series of 130 CM, among which were comprised the cases previously matched for poly(ADP-ribosyl)ation. The aim of the study was to verify the hypothesis that the metastasizing behavior of CM could be correlated with the alteration of more than a single epigenetic pathway of DNA-repair and cell proliferation control, and to establish the possible role of CAF-1/p60 as a new valuable predictor of prognosis for skin melanoma patients.

## Methods

### Study population

Formalin-fixed, paraffinized blocks of histologically confirmed primary CM were selected from the archive files of the Department of Biomorphological and Functional Sciences, Pathology Section, University Federico II of Naples, among all the cases of skin melanomas surgically excised at the Dermatology Section and Plastic Surgery of the Department of Systematic Pathology of the same Institution between January 1985 and December 2007. Inclusion criteria were: (i) primary melanomas that have arisen in photoexposed skin; (ii) absence of multiple lesions, hereditary history of skin cancer, and/or exposition to physical and/or chemical predisposing factors; (iii) post-surgical clinical follow-up, covering a period ranging from 1 to 22 years. Following the above described selection criteria, 130 cases of primary CM have been considered suitable for the study.

The study population consisted of 64 men and 66 women, with an average age of 47.02 years (range 17-84 years) (Table 1). The depth of vertical invasion (Breslow thickness) of CM was lesser than 1.00 mm in 28 (21.6%) cases, ranged from 1.01 and 2.00 mm in 51 cases (39.2%), from 2.01 and 4.00 mm in 41 (31.5%), and was deeper than 4.00 mm in 10 cases (7.7%) (Table 1). In all cases, CM tissue derived from routine excision with safety margins. Ulceration was found in 36 CM.

**Table 1:** Clinical and pathological features of the study population of CM (ordered by Breslow depth, 130 total cases)

Breslow*	Sex (M/F)	Age (yrs)	Ulceration	Follow-up (yrs)	Clinical outcome
**≤/= 1.00 mm**	16/12 (N° = 28)	46,1 (22-74)	2	11,3 (2-21)	1S; 1N

**1.01 - 2.00 mm**	24/27 (N° = 51)	47,6 (18-84)	9	9,1 (2-22)	1S; 4N; 1S, N; 1N, M; 1N, M, D

**2.01-4.00 mm**	19/22 (N° = 41)	45,8 (17-81)	16	7,6 (1-14)	10N; 3M; 2N, M; 4N, M, D

**>4.00 mm**	4/6 (N° = 10)	51,4 (30-81)	9	6.3 (1-23)	3N; 1N, M; 1S, M, D; 1N, M, D

* Breslow depth classes were determined according to AJCC system (2009)^10^

S: skin (dermal/hypodermal) metastasis; N: nodal metastasis; M: distant metastasis; D: death for disease

The follow-up was available for all patients (mean follow-up: 9.01 years, range: 1-22 years).

Two patients (1.5%) developed skin (dermal/hypodermal) metastasis during follow-up; 18 patients (13,8%) had node metastasis, and 3 developed distant metastases (2,3%); one patient (0,8%) experienced either nodal and skin metastasis, and one patient (0,8%) developed skin and distant metastases. Ten patients (7,7%) developed both nodal and distant metastases. (Table 1)

Histopathological features and case histories have been recorded in a standardized manner, and included age, gender, tumor thickness (Breslow depth classes, according to the American Joint Committee on Cancer, AJCC 2009), and the date of diagnosis [10].

The study was performed according to the guidelines of the Institutional Ethic Committee, which, in agreement with the Italian law, with reference to the topics of the present research, do not provide for the Ethical committee approval, and, according to the Declaration of Helsinki require, for studies based on retrospective analyses on routine archival formalin-fixed, paraffin embedded tissue, only a written informed consent from the alive patient, following the indication of Italian DLgs n° 196/03 (Codex on Privacy) at the time of Surgery for the primary melanoma.

### Immunohistochemistry

For each case, 4-μm-thick serial sections have been cut and mounted on poly-L-lysine coated glass slides. For hyperpigmented tumors, bleaching of melanin was achieved by incubating the sections in a solution of 0.25% potassium permanganate, 5% oxalic acid, for 60 minutes at room temperature. Paraffinized tissue blocks of human normal skin from 10 patients that underwent reconstructive surgery for non-neoplastic pathologies, and 15 tissue blocks of benign melanocytic naevi (5 junctional, 5 compound and 5 intradermal) were used as standard control for the expression of CAF-1/p60 respectively in normal melanocytes and in benign melanocytic tumors.

**Figure 1:**
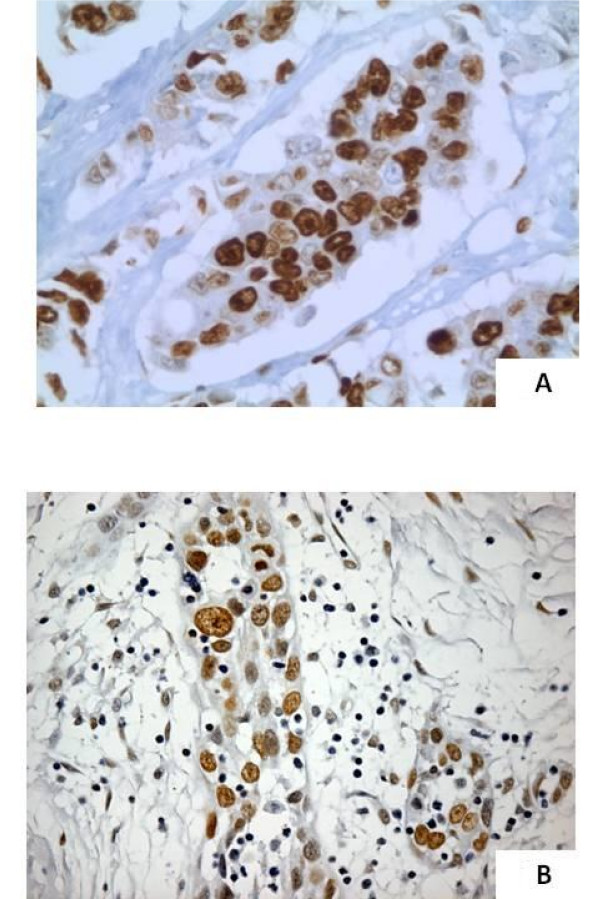
**Positive controls for CAF-1/p60 immunostaining**. A case of breast carcinoma (1A, ×400) and a case of squamous cell carcinoma (1B, ×400) showing a strong expression of CAF-1/p60.

Deparaffinized sections of all cases were boiled three times for 3 min in a 10^-3 ^M sodium citrate buffer (pH 6.0) as antigen retrieval method. In order to prevent the non-specific bindings of the antibody, the sections were pre-incubated with non-immune mouse serum (1:20, Dakopatts, Hamburg, Germany) diluted in PBS/BSA, 1%, for 25 minutes, at room temperature. After quenching of endogenous peroxidases with 0.3% hydrogen peroxide in methanol, followed by two rinses with Tris-HCl buffer, the sections were incubated, overnight at 4°C, with the anti-CAF-1/p60 antibody (SS53 - ab8133, Abcam, Cambridge, MA, USA, as previously described), diluted 1:300 [30,31]. The standard streptavidin-biotin-peroxidase complex technique was performed, using sequential 20-minutes incubation with biotin-labeled secondary antibody (1:30) and with peroxidase-labelled streptavidin (1:30) for 10 minutes (DAKO LSAB kit HRP, Carpinteria, CA). For the development of the peroxidase activity, 3, 3'-diaminobenzidine (DAB, Vector Laboratories, Burlingame, U.S.A.) was used as a substrate chromogen solution. Haematoxylin was used for nuclear counterstaining, then the sections were mounted and cover-slipped with a synthetic mounting medium (Entellan, Merck, Germany). For each staining run were used as positive controls sections from breast cancer and oral squamous cell carcinoma [30,31]. Only cells with a definite brown nuclear staining were judged positive for the antibody (Figure 1A, B). As previously described, the immunohistochemical expression was evaluated as percentage of positive tumour cells among the total neoplastic cells present in at least 10 high power fields. The expression of CAF-1/p60 was then quantified semiquantitatively according to an arbitrary scale, as follows: 0 (<10% of positive cells); + (10% - < 20%); ++ (20% - <30%); +++ (≥ 30% of positive cells) [31,32]. Negative controls were performed substituting the primary antibody with a mouse myeloma protein of the same subclass, at the same concentration. Slides were evaluated blindly by two observers (SS and MM) and the cases with discordance were discussed and resolved by consensus.

### Statistical analysis

Data have been analyzed using the SPSS (version 13.0) package for Windows and with software for Microsoft Excel (a P-value of < 0.05). Univariate analysis of non-parametric variables was performed by Spearman's non- parametric correlation test. Disease-free survival (DFS) was calculated from the date of surgery for the primary melanoma to the date of the occurrence of the first loco-regional (dermal/hypodermal or nodal) and/or distant metastasis. Death was scored as an event, and alive patients were censored at the time of the last follow-up. DFS curves were drawn using Kaplan-Meier estimates and were compared using log-rank test. Survival rates were presented with their 95% confidence intervals. Multinomial logistic regression was used to evaluate the role of sex, age of patients, Breslow thickness, ulceration and CAF-1/p60 expression in radial and vertical growth phases of CM as predictor variables of unfavorable outcome of the tumors

The prognostic accuracy of the selected predictor variables was tested by the receiver operating characteristics (ROC) curve, in relation with the more significant prognostic parameters. The area under the ROC curve has been utilized to measure the discriminatory ability of each parameter, as follows: < 0,7: no discrimination; 0,71-0,79: acceptable; 0,8-0,89: excellent; ≥ 0,9: outstanding discrimination [33]

## Results

### Patients

As expected, DFS of patients was influenced by tumor thickness. At the end of the follow-up, among the 35 cases of CM with an unfavorable follow-up, the Breslow depth was < 1.0 mm in two cases (1.5%), comprised between 1.01 and 2.00 mm in 8 cases (6.2%), between 2.01 and 4.00 mm in 19 cases (14.6%), and >4.00 mm in 6 CM (4.6%). Among these patients, 7 (20%) died (one with cutaneous and distant metastases within 12 months from the diagnosis, and six with nodal and distant metastases, respectively after 7 and 60, 11 and 48, 8 and 44, 12 and 22, 5 and 12, 3 and 4 months from the diagnosis). (Table 1)

The percentage of cases with metastatic behavior among each Breslow group of tumors varied as follows: 60% (6/10 patients) for CM deeper than 4.00 mm, 46.3% (19/41 patients) for CM from 2.01 to 4.00 mm, 15.68% (8/51 patients) for the melanomas between 1.01 and 2.0 mm, and 7.1% (2/28 patients) for CM thinner than 1.00 mm.

### CAF-1 expression in normal melanocytes

In normal skin specimens, only a few melanocytes at the dermal-epidermal junction showed low (+) nuclear expression of CAF-1/p60.

### CAF-1 expression in melanocytic naevi

Nuclear immunoreactivity for CAF-1/p60 was found in up to 5% (low expression: +) of melanocytes, mainly located at the junctional and superficial dermal levels. Deep melanocytes showed, almost invariably, absence of immunohistochemical expression of the protein.

### CAF-1 expression in cutaneous melanoma

**Figure 2:**
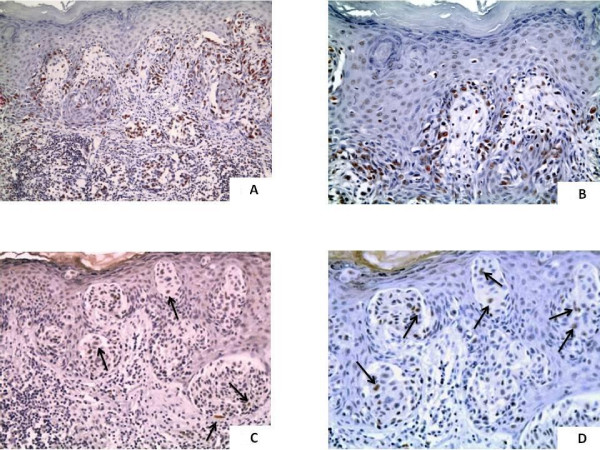
**CAF-1 expression in "thin" cutaneous melanoma**. A case of primary "thin" cutaneous melanoma (< 1 mm Breslow depth) showing over-expression of CAF-1/p60. This patient experienced node metastasis 7 years from diagnosis (2A: original magnification, ×150; 2B: ×200); "Thin" cutaneous melanoma, disease-free 11 years from surgery: low expression of CAF-1/p60; (2C: original magnification, ×200; 2D, ×250). Arrows indicate the immunostained melanocytes.

**Figure 3:**
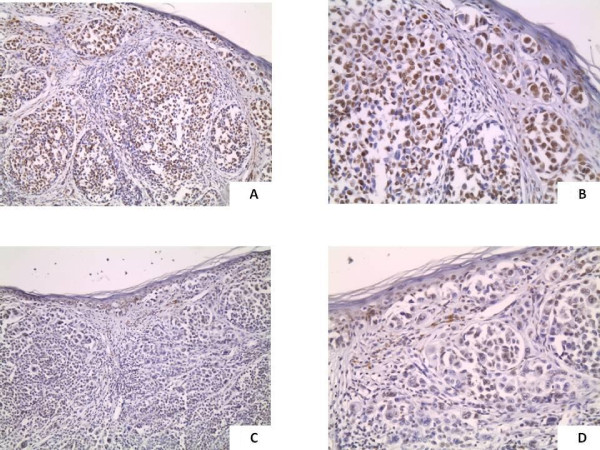
**CAF-1 expression in thick cutaneous melanoma**. Thick cutaneous melanoma (Breslow thickness: > 4 mm) with diffuse over-expression of CAF-1/p60. This patient developed nodal and distant metastases and died for disease 2 years from diagnosis (3A: original magnification, ×150; 3B, ×200); Thick cutaneous melanoma, disease-free 12 years after surgery: moderate expression of CAF-1/p60 (3C: original magnification, ×150; 3D, ×200).

All the cases of CM showed nuclear expression of CAF-1/p60 in cells of both the radial (intraepithelial) growth phase and vertical (invasive) growth phase. The highest level of expression (+++) of CAF-1/p60 was found only in malignant melanocytes of the vertical growth phase (in 37 cases); a moderate level of expression (++) was found in 46 cases of vertical phase CM and in the radial growth phase of 39 melanomas; a low expression level (+) was found in the radial growth phase of the remaining 91 CM (Figures 2, 3) (Table 2).

**Table 2:** Clinical and pathological features and CAF-1/p60 immunohistochemical expression of 130 cases of CM ordered by Breslow depth

Case	Age (y)	Sex	Breslow	Ulceration	Mitosis	p60 r (%)	p60 r	p60 v (%)	p60 v	Follow-up (yrs)
1*	58	F	≤/= 1.00	no	< 1	10	+	17	+	12

2*	36	M	≤/= 1.00	no	≥ 1	11	+	15	+	12

3*	67	M	≤/= 1.00	no	< 1	10	+	18	+	12

4*	45	F	≤/= 1.00	no	≥ 1	10	+	13	+	12

5*	50	F	≤/= 1.00	no	< 1	12	+	15	+	11

6*	49	F	≤/= 1.00	no	≥ 1	14	+	18	+	11

7*	42	M	≤/= 1.00	no	< 1	10	+	14	+	10

8*	43	F	≤/= 1.00	no	≥ 1	12	+	14	+	9

9*	43	M	≤/= 1.00	no	< 1	13	+	16	+	9

10*	49	M	≤/= 1.00	no	≥ 1	28	++	37	+++	7

11*	40	M	≤/= 1.00	no	≥ 1	18	+	22	++	7

12*	41	M	≤/= 1.00	yes	< 1	15	+	25	++	7

13*	39	M	≤/= 1.00	no	< 1	27	++	45	+++	6 S (52 m**)

14*	37	F	≤/= 1.00	no	≥ 1	17	+	28	++	6

15	42	M	≤/= 1.00	no	< 1	12	+	16	+	14

16	37	M	≤/= 1.00	no	< 1	13	+	19	+	19

17	67	M	≤/= 1.00	no	≥ 1	11	+	14	+	14

18	66	M	≤/= 1.00	no	< 1	11	+	14	+	17

19	56	M	≤/= 1.00	no	≥ 1	10	+	23	++	2

20	35	M	≤/= 1.00	yes	≥ 1	15	+	20	++	7

21	32	F	≤/= 1.00	no	< 1	14	+	22	++	6

22	73	M	≤/= 1.00	no	≥ 1	10	+	14	+	21

23	74	F	≤/= 1.00	no	< 1	12	+	16	+	21

24	41	F	≤/= 1.00	no	≥ 1	10	+	14	+	14

25	48	F	≤/= 1.00	no	< 1	11	+	15	+	14

26	22	M	≤/= 1.00	no	≥ 1	12	+	15	+	14

27	24	F	≤/= 1.00	no	< 1	13	+	17	+	15

28	36	F	≤/= 1.00	no	≥ 1	28	++	50	+++	4 N (7 m)

29*	66	F	1.01 - 2.00	no	≥ 1	26	++	70	+++	12 N (8 m)

30*	65	F	1.01 - 2.00	no	< 1	20	++	24	++	12

31*	56	M	1.01 - 2.00	yes	≥ 1	21	++	26	++	12

32*	49	M	1.01 - 2.00	no	< 1	12	+	14	+	12

33*	48	M	1.01 - 2.00	no	≥ 1	11	+	15	+	12

34*	39	F	1.01 - 2.00	no	< 1	17	+	26	++	12

35*	47	F	1.01 - 2.00	yes	≥ 1	25	++	70	+++	12 N (7 m), M(60 m), D(144 m)

36*	51	M	1.01 - 2.00	no	< 1	10	+	22	++	11

37*	53	M	1.01 - 2.00	no	≥ 1	10	+	14	+	11

38*	56	M	1.01 - 2.00	no	< 1	11	+	14	+	11

39*	51	F	1.01 - 2.00	no	≥ 1	18	+	21	++	10

40*	45	F	1.01 - 2.00	no	< 1	10	+	15	+	10

41*	53	M	1.01 - 2.00	no	≥ 1	11	+	15	+	10

42*	67	M	1.01 - 2.00	no	< 1	12	+	14	+	10

43*	54	M	1.01 - 2.00	no	≥ 1	10	+	15	+	10

44*	34	M	1.01 - 2.00	no	≥ 1	13	+	22	++	9

45*	44	F	1.01 - 2.00	no	< 1	14	+	24	++	9

46*	43	F	1.01 - 2.00	no	< 1	11	+	23	++	9

47*	71	F	1.01 - 2.00	no	≥ 1	10	+	23	++	9

48*	34	M	1.01 - 2.00	no	< 1	15	+	20	++	9

49*	43	F	1.01 - 2.00	no	≥ 1	10	+	25	++	9

50*	51	F	1.01 - 2.00	no	< 1	12	+	14	+	9

51*	50	M	1.01 - 2.00	no	< 1	10	+	14	+	9

52*	39	F	1.01 - 2.00	no	< 1	25	++	40	+++	9 N (75 m)

53*	29	F	1.01 - 2.00	no	≥ 1	13	+	14	+	9

54*	42	M	1.01 - 2.00	no	< 1	11	+	14	+	7

55*	37	F	1.01 - 2.00	yes	≥ 1	27	++	50	+++	7 S (12 m), N(7 m)

56*	65	M	1.01 - 2.00	no	< 1	10	+	14	+	7

57*	43	M	1.01 - 2.00	no	≥ 1	23	++	45	+++	7 N(9 m)

58*	44	F	1.01 - 2.00	no	< 1	10	+	28	++	6

59*	18	F	1.01 - 2.00	no	≥ 1	15	+	27	++	4

60*	22	M	1.01 - 2.00	no	≥ 1	14	+	25	++	4

61*	24	M	1.01 - 2.00	no	< 1	12	+	22	++	4

62*	32	M	1.01 - 2.00	no	< 1	10	+	23	++	4

63*	30	F	1.01 - 2.00	no	< 1	12	+	21	++	4

64*	38	F	1.01 - 2.00	no	≥ 1	14	+	24	++	4

65*	37	M	1.01 - 2.00	no	< 1	15	+	23	++	3

66*	40	M	1.01 - 2.00	no	≥ 1	10	+	22	++	3

67	51	M	1.01 - 2.00	no	< 1	13	+	16	+	22

68	34	F	1.01 - 2.00	no	≥ 1	10	+	14	+	14

69	66	F	1.01 - 2.00	no	< 1	12	+	14	+	15

70	60	M	1.01 - 2.00	yes	< 1	11	+	14	+	13

71	48	F	1.01 - 2.00	no	≥ 1	10	+	14	+	14

72	28	M	1.01 - 2.00	yes	< 1	24	++	58	+++	2 N(3 m)

73	55	F	1.01 - 2.00	no	≥ 1	12	+	16	+	19

74	58	F	1.01 - 2.00	yes	≥ 1	28	++	50	+++	4 S

75	32	F	1.01 - 2.00	no	< 1	10	+	22	++	4

76	84	F	1.01 - 2.00	no	≥ 1	12	+	27	++	14

77	66	M	1.01 - 2.00	yes	≥ 1	26	++	47	+++	14 N(5,6 m), M(9,25,32 m)

78	75	F	1.01 - 2.00	yes	≥ 1	12	+	14	+	14

79	64	F	1.01 - 2.00	no	< 1	10	+	14	+	12

80*	40	M	2.01-4.00	no	< 1	11	+	14	+	12

81*	36	M	2.01-4.00	no	≥ 1	14	+	14	+	12

82*	39	M	2.01-4.00	no	< 1	10	+	22	++	11

83*	50	F	2.01-4.00	no	≥ 1	10	+	21	++	11

84*	48	F	2.01-4.00	no	< 1	14	+	25	++	11

85*	45	M	2.01-4.00	yes	≥ 1	27	++	57	+++	11 N(11 m), M(48 m), D(132 m)

86*	43	F	2.01-4.00	no	≥ 1	11	+	14	+	10

87*	53	F	2.01-4.00	no	< 1	15	+	25	++	10

88*	47	M	2.01-4.00	no	< 1	26	++	43	+++	10 N(3 m)

89*	54	M	2.01-4.00	no	≥ 1	12	+	24	++	10

90*	50	M	2.01-4.00	no	< 1	14	+	19	+	10

91*	69	F	2.01-4.00	no	≥ 1	12	+	14	+	10

92*	54	M	2.01-4.00	no	< 1	10	+	14	+	10

93*	53	M	2.01-4.00	no	< 1	10	+	22	++	9

94*	42	F	2.01-4.00	yes	≥ 1	25	++	68	+++	9 N(8 m), M(44 m), D(108 m)

95*	37	F	2.01-4.00	yes	≥ 1	26	++	60	+++	9 N(5 m), M(32 m)

96*	45	F	2.01-4.00	no	< 1	12	+	14	+	9

97*	49	F	2.01-4.00	yes	≥ 1	27	++	59	+++	9 N(12 m), M(22 m), D(108 m)

98*	32	M	2.01-4.00	yes	≥ 1	25	++	65	+++	7 N(10 m)

99*	38	F	2.01-4.00	yes	≥ 1	28	++	68	+++	6 N(10 m)

100*	38	M	2.01-4.00	no	< 1	12	+	25	++	3

101*	32	M	2.01-4.00	yes	≥ 1	29	++	60	+++	3 N(12 m), M(25 m)

102*	40	M	2.01-4.00	no	< 1	13	+	23	++	3

103*	46	F	2.01-4.00	no	≥ 1	24	++	65	+++	3 N(9 m)

104*	50	M	2.01-4.00	no	< 1	12	+	24	++	2

105*	17	M	2.01-4.00	no	< 1	22	++	55	+++	12 N(84 m)

106*	30	M	2.01-4.00	no	≥ 1	15	+	25	++	2

107*	32	F	2.01-4.00	no	< 1	20	++	36	+++	2

108	81	F	2.01-4.00	yes	≥ 1	22	++	50	+++	2 M(17 m)

109	56	F	2.01-4.00	no	< 1	12	+	27	++	13

110	22	F	2.01-4.00	yes	< 1	24	++	65	+++	2 N(3 m)

111	21	M	2.01-4.00	yes	< 1	14	+	22	++	14

112	60	F	2.01-4.00	no	≥ 1	25	++	60	+++	1 N(4 m)

113	33	F	2.01-4.00	yes	< 1	24	++	65	+++	1 M(8 m)

114	60	M	2.01-4.00	no	≥ 1	15	+	23	++	13

115	73	F	2.01-4.00	yes	< 1	22	++	70	+++	12 N(12 m)

116	42	M	2.01-4.00	yes	≥ 1	26	++	68	+++	6 N(6 m)

117	45	M	2.01-4.00	yes	< 1	25	++	63	+++	4 M(26,28 m)

118	56	F	2.01-4.00	no	≥ 1	24	++	70	+++	13 N(5 m)

119	53	F	2.01-4.00	yes	< 1	16	+	23	++	12

120	65	F	2.01-4.00	yes	≥ 1	26	++	65	+++	2 N(5 m)M(12 m)D(24 m)

121	38	F	>4.00	yes	< 1	24	++	60	+++	1 N(2 m)

122	55	F	>4.00	yes	< 1	17	+	21	++	14

123	56	F	>4.00	yes	< 1	23	++	68	+++	11 N(3 m), M(24 m)

124	69	M	>4.00	yes	≥ 1	18	+	22	++	6

125	81	F	>4.00	yes	< 1	24	++	58	+++	1 N(12 m)

126	52	F	>4.00	yes	< 1	28	++	70	+++	2 S(12 m), M(12 m), D(24 m)

127	30	F	>4.00	yes	≥ 1	29	++	63	+++	2 N(3 m), M(4 m), D(24 m)

128	54	M	>4.00	no	< 1	15	+	24	++	12

129	44	M	>4.00	yes	≥ 1	27	++	70	+++	12 N(3 m)

130	35	M	>4.00	yes	≥ 1	12	+	25	++	2

S: skin (dermal/hypodermal) metastasis; N: nodal metastasis; M: distant metastasis; D: death for disease; °P60r: CAF-1/P60 in radial growth phase; °°P60v: CAF-1/P60 in vertical growth phase; (*): cases previously matched for PARP-1 (poly-ADP-ribosyl)ase-1 expression; ** m = months.

The cases of CM with the strongest CAF-1/p60 expression in vertical phase comprised all the 15 cases in which previously an alteration of the poly-(ADP-ribosil)ation process has been evidenced [18] (Table 2).

The univariate statistic analysis showed a correlation between a moderate (++) level of CAF-1/p60 expression in radial growth phase and a high (+++) expression of the protein in the vertical growth phase of CM and sex of patients.

A P < 0,001 level of significance resulted from the correlation between a ++ expression of CAF-1/p60 in radial growth phase, and a +++ expression in vertical growth phase of CM and the Breslow depth, staging, recurrence and/or death for disease (Tables 3,4). Table 5 shows the detailed expression of CAF-1/p60 according to the Breslow thickness.

**Table 3:** Clinic and pathologic findings in 130 patients with CM, according to CAF-1/p60 expression in radial and vertical growth phase.

CLINICO-PATHOLOGIC FEATURES		CAF-1/P60 radial growth phase							CAF-1/P60 vertical growth phase						
	**N°**	**+**	**%**	**++**	**%**	**+++**	**%**	**PVALUE°**	**+**	**%**	**++**	**%**	**+++**	**%**	**PVALUE°**

**AGE**															

≤30	13	9	69,2%	4	30,8%	0	0	NS	3	23,0%	6	46,2%	4	30,8%	NS

>30	117	82	70,0%	35	30%	0	0	NS	44	37,6%	40	34,2%	33	28,2%	NS

															

**STAGING**															

IA	25	22	88,0%	3	12,0%	0	0	<0,001	19	76,0%	3	12,0%	3	12,0%	<0,001

IB	45	42	93,3%	3	6,7%	0	0	<0,001	19	42,2%	24	53,3%	2	4,5%	<0,001

IIA	28	20	71,4%	8	28,6%	0	0	<0,001	9	32,1%	12	42,8%	7	25,1%	<0,001

IIB	16	3	18,7%	13	81,3%	0	0	<0,001	0	0	3	18,7%	13	81,3%	<0,001

IIC	4	3	75,0%	1	25,0%	0	0	<0,001	0	0	1	25,0%	3	75,0%	<0,001

IIIA	4	1	25,0%	3	75,0%	0	0	<0,001	0	0	1	25,0%	3	75,0%	<0,001

IIIB	7	0	0	7	100%	0	0	<0,001	0	0	0	0	7	100%	<0,001

IV	1	0	0	1	100%	0	0	<0,001	0	0	0	0	1	100%	<0,001

															

**BRESLOW**															

<1 mm	28	25	89,3%	3	10,7%	0	0	<0,001	19	67,8%	6	21,4%	3	10,8%	<0,001

1.01-2.00 mm	51	41	80,4%	10	19,6%	0	0	<0,001	21	41,2%	22	43,1%	8	15,7%	<0,001

2.01-4.00 mm	41	21	51,2%	20	48,8%	0	0	<0,001	7	17,1%	14	34,1%	20	48,8%	<0,001

>4.00 mm	10	4	40,0%	6	60,0%	0	0	<0,001	0	0	4	40,0%	6	60,0%	<0,001

															

**PROGNOSIS**															

NO PROGRESSION	95	91	95,8%	4	4,2%	0	0	<0,001	47	49,5%	46	48,4%	2	2,1%	<0,001

S/N/M*	35	0	0	35	100%	0	0	<0,001	0	0	0	0	35	100%	<0,001

															

*S: skin (dermal/hypodermal) metastasis; N: nodal metastasis; M: distant metastasis; °(P value were calculated by t-test or ANOVA)

**Table 4:** Spearman's correlation coefficient between all variables analyzed.

	Breslow	P60r*	P60v**	Staging
Breslow/Correlation coefficient	-	,369	,386	,909

Sig. (2-tailed)	-	,000	,000	,000

Radial p60/Correlation coefficient	,369	-	,963	,544

Sig. (2-tailed)	,000	-	,000	,000

Vertical p60/Correlation coefficient	,386	,963	-	,552

Sig. (2-tailed)	,000	,000	-	,000

Staging/Correlation coefficient	,909	,544	,552	-

Sig. (2-tailed)	,000	,000	,000	-

*P60r: CAF-1/P60 in radial growth phase; **60v: CAF-1/P60 in vertical growth phase

**Table 5:** CAF-1/p60 expression in 130 primary melanomas subdivided according to Breslow thickness

Melanoma thickness	Patients	CAF-1/P60 radial growth phase						CAF-1/P60 vertical growth phase					
	N°	+	%	++	%	+++	%	+	%	++	%	+++	%
**≤ 1 mm**	28	25	19,2%	3	2,3%	0	0	19	14,6%	6	4,6%	3	2,3%
**1.01-2 mm**	51	41	31,6%	10	7,7%	0	0	21	16,1%	22	16,9%	8	6,2%
**2.01-4 mm**	41	21	16,1%	20	15,4%	0	0	7	5,4%	14	10,8%	20	15,4%
**>4 mm**	10	4	3,1%	6	4,6%	0	0	0	0	4	3,1%	6	4,6%

**Figure 4:**
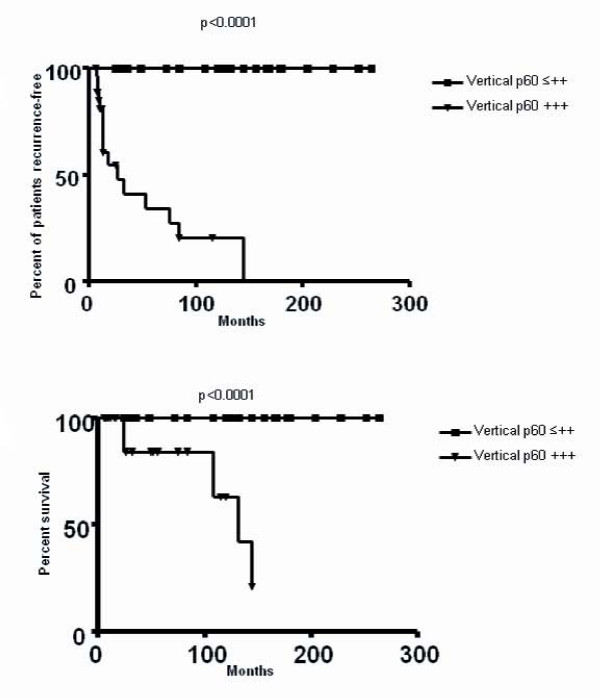
**Log-rank testing analysis**. The incidence of recurrence (4A) and death for disease (4B) of patients is significantly associated with the highest level (+++) of CAF-1/p60 expression in the vertical phase of CM (P = 0,001);

Moreover, the analysis with log-rank testing showed that the incidence of recurrence and death for disease of patients was significantly associated (P = 0,001) with the highest level (+++) of CAF-1/p60 expression in the vertical phase of CM (Figure 4A, B)

The multivariate analysis (logistic regression analysis), revealed that only high expression (+++) of CAF-1/p60 in vertical growth phase and staging had predictive significance in respect to recurrence of melanomas (p < 0,0001 and p < 0,0001, respectively).

The prognostic accuracy of these two parameters (CAF-1/p60 +++ hyperexpression in vertical growth phase of CM and staging at the time of diagnosis) was tested by the receiver operating characteristic (ROC) curve. In detail, patients were subdivided into 4 categories according to CAF-1/p60 expression (**1**: < 10%, **2: **11-20%, **3: **21-30%, **4: **>30% positive cells) and into 8 categories according to staging sec. AJCC (stage groups: 0, IA, IB, IIA, IIB, IIC, III, IV).

**Figure 5:**
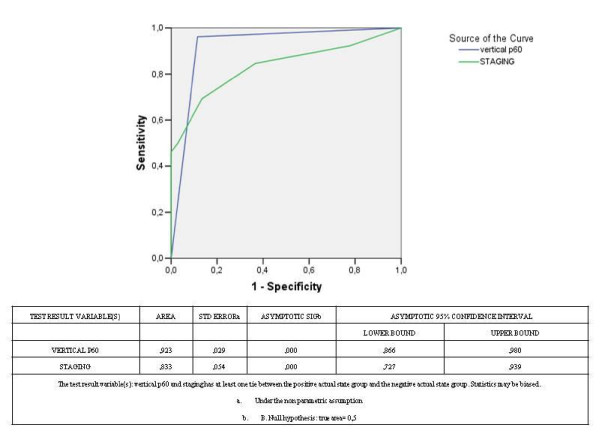
**Receiver operating characteristic (ROC) curve**. ROC curve evidence an excellent performance of CAF-1/p60 +++ expression level in the vertical phase both in terms of sensitivity and specificity (area under curve: 0,92).

Both parameters were able to accurately predict the clinical outcome of CM patients even if the diagnostic accuracy of CAF-1/p60 +++ expression level in the vertical phase both was significantly better than that of staging (area under curve: 0,92 and 0.83, respectively, Figure 5).

Both parameters were able to accurately predict the clinical outcome of CM patients even if the diagnostic accuracy of CAF-1/p60 +++ expression level in the vertical phase both was significantly better than that of staging (area under curve: 0,92 and 0.83, respectively).

## Discussion

As it is known, despite its location within the protective chromatin microenvironment, DNA is subject to repeated damage by exogenous agents, such as ultraviolet radiation (UV), which can impair replication and/or transcription and must be corrected to prevent mutations [24].

Chromatin dynamics tightly controls activation and function of genes, by modulating the access of regulatory factors to DNA, and highly influences cellular behavior of normal and neoplastic cells [21]. A highly ordered coordination and organization of factors regulating the opening and closing of chromatin is crucial to ensure that the correct epigenetic code is maintained within the genome [24].

Genomic instability is a major hallmark of tumor progression. It contributes to the gain of the invasive-metastatic phenotype of neoplastic cells by favoring the accumulation of sequential genetic and epigenetic events. These latter include DNA methylation, histone modifications, small, non-coding RNAs, and factors involved in the regulation of chromatin architecture [34-36].

Chromatin dynamics control nucleosome assembly and higher order chromatin structure, and are responsible for the global nuclear organization and compartmentalization, tightly regulating gene expression and controlling protein-DNA interactions within the cell nucleus [37,38]. In particular, it allows the recognition of DNA-damaged sequences and the segregation of the damaged chromosomes, potential cause of genomic instability, determining chromatin reshaping and warranting the correct progression of the cell cycle and the maintenance of genome integrity [39-41].

In melanomagenesis, the deregulation of cell proliferation results, at least in part, from the alteration of epigenetic control checkpoints. In particular, the inactivation of UV-specific pathways of DNA repair is a critical step.

In normal melanocytes, UV trigger histone acetylation and the immediate activation of the histone poly-(ADP-ribosyl)ation, a post-synthetic DNA epigenetic modification which causes the generation of intracellular signals, leading to the DNA repair or, in the case of excessive damage, to apoptosis [24,41-43].

Recent data concerning the "chromatin ambient" of the nucleus indicate a highly qualified role for chromatin modifying proteins in the control of DNA replication and DNA-repair [27,44,45]. The CAF-1 molecular complex looks fundamental for the maintenance of the epigenetic information; in particular, the deletion of CAF-1 subunits in S. cerevisiae confers sensibility to UV radiation [24]. CAF-1, to date, is the only known chromatin assembly factor able to drive nucleosome assembly onto newly synthesized DNA, and has a central role in ensuring chromatin replication in S phase through the interaction with the polymerase sliding clamp, PCNA [26,30,31,46-48]. It constitutes the first example of a factor involved in chromatin dynamics useful to assess cell proliferation [30]. The behavior of its p60 and p150 subunits shares a number of similarities with PCNA, which plays crucial roles in both DNA replication and repair. Experimental data indicate that particularly p60 is active in either the control of cell proliferation and DNA-repair. Silencing of the p60 subunit by RNAi leads to the accumulation of double-strand DNA breaks and to the induction of programmed cell death in proliferating but not quiescent human cells [49].

Recently, the involvement of CAF-1/p60 in neoplastic progression has been reported, and its expression has been proposed as a new tool to define the biological behavior of some types of human malignancies (breast, tongue, and prostate cancer) [30-32].

This opens up the possibility that its use could be extended to malignancies of different histogenesis.

Here we show results supporting a role of CAF-1 in predicting the aggressiveness of CM.

In our cases, the maximum level of over-expression of CAF-1/p60 was found in the vertical growth phase of CM characterized by a metastasizing behavior, besides the Breslow thickness, ulceration and/or high mitotic index. This suggests that CAF-1/p60 may shows a promising role as a relevant, adjunctive prognostic marker for CM. Our results are in-line with previous data concerning the alteration of the poly-(ADP-ribosil)ation process in highly aggressive CM [18]. In particular, the occurrence of CAF-1/p60 hyperexpression in the same cases of CM previously found to bear an altered expression of PARP-1, indicates that the co-existence of the deregulation of two of the major epigenetic pathways responsible of DNA-repair and cell replication control, constitutes a hallmark predictive of metastasizing behavior in CM of photoexposed skin.

Investigations are being undertaken from our research group to determine whether the alteration of such mechanisms still retains with the same statistical significance among a multi-institutional greater series of cases. This perspective looks extremely attractive, because give us means to built up a model in which the evaluation of the CAF-1/p60 status of expression could get over the limitations of current prognostic evaluation, particularly for that concerning the "grey area" of CM with an intermediate thickness (Breslow >1 and <2 mm).

In addition, it would be worth investigating how the CAF-1 molecular pathway can be regulated, by modulating the expression of its subunits and their interactions with the other histone chaperones and ATP-dependent chromatin remodelers. It has to be remembered, to this regard, that in contrast to genetic alterations, epigenetic modifications, although heritable in cells, are progressive, quantitatively evaluable, potentially reversible, and may serve as potential targets for drug treatment [22,50,51]. Given the absence of effective therapy for metastatic melanoma, the CAF-1 pathway may provide a window of opportunity for novel post-surgical molecular therapies. Undoubtedly, future studies will provide many exciting advances towards fully understanding of the critical link between chromatin, DNA repair and cell proliferation control, and the biology of CM.

Considering its essential role for cell cycle progression, it might constitute a new therapeutic target with cytostatic and/or cytotoxic effects that deserve to be investigated in the future.

Basing on the results of the present study, however, we believe that CAF-1/p60 expression is an exciting chance to better predict the biological behavior of CM, besides the traditional prognostic parameters.

## Conclusions

This paper constitutes the first report of CAF-1/p60 up-regulation in CM, and supports a noteworthy role for CAF-1 in linking chromatin dynamics to the metastasizing behavior of this tumor.

Basing on results, CAF-1 looks like a promising candidate as a new prognostic marker which might give us the chance to early detect the subset of CM patients affected by metastasizing, hence deadly tumors, which cannot be correctly detected on the basis of the existing traditional parameters.

Many interesting questions remain still unanswered about how chromatin remodeling influences replication, transcription and repair within the tumorigenesis cascade of CM. However, according to our opinion, our findings have opened up new intriguing horizons that may enable a better understanding of the biology of skin melanoma, providing further insights into the contribution of the epigenetic control of cell proliferation and DNA repair to the gain of a metastasizing phenotype of CM.

## Competing interests section

The authors declare that they have no competing interests.

## Authors' contributions

MM participated in conception and design, analysis and interpretation of data and drafting of manuscript. *MLV participated in analysis and interpretation of data and acquisition and processing of images/microphotographs. *GI carried out immunostaining of tissue section and participated in analysis of data and drafting of the materials and methods section. MS participated in collection of study population (skin biopsies and sentinel node), acquisition of clinical data and clinical follow-up of patients. GM participated in collection of study population (skin biopsies and sentinel node), acquisition of clinical data and clinical follow-up of patients. MDB participated in statistical analysis and interpretation of data. LN participated in acquisition of data and drafting of the results section. *MS participated in analysis and interpretation of data and acquisition and processing of images/microphotographs. GDR participated in critical analysis of results and manuscript revision. SS participated in conception and design, analysis and interpretation of data, drafting and critical revision of the manuscript. All authors read and approved the final manuscript.

## Pre-publication history

The pre-publication history for this paper can be accessed here:

http://www.biomedcentral.com/1471-2407/10/63/prepub
